# Functional outcomes of patients in ICU using the Chelsea Critical Care Physical Assessment tool: An integrative review

**DOI:** 10.4102/sajp.v79i1.1924

**Published:** 2023-11-16

**Authors:** Lebogang C. Tjale, Silmara G. Hanekom, Nombeko Mshunqane

**Affiliations:** 1 Department of Physiotherapy, Faculty of Health, University of Pretoria, Pretoria, South Africa; 2 Department of Physiotherapy, Faculty of Health, University of the Free State, Bloemfontein, South Africa

**Keywords:** critically ill patients, functional outcomes, intensive care unit, Chelsea Critical Care Physical Assessment tool, physiotherapy, outcome measures, physical functions

## Abstract

**Background:**

Outcome measures can assess the change in the health status of a patient in an intensive care unit (ICU). The Chelsea Critical Care Physical Assessment (CPAx) tool is used to assess the functional outcomes to monitor patient progression or regression in an ICU.

**Objectives:**

Our study aimed to identify studies that assess the functional outcomes of patients nursed in ICUs that use the CPAx tool.

**Method:**

An integrative review framework was used. Data were analysed in five steps to formulate a conclusion that aligned with the objective of our study. Data were extracted from peer-reviewed articles published online between 2013 and 2022. Databases that were used include Google Scholar, Directory of Open Access Journals (DOAJ) and PubMed for reviewed articles. Keywords were used in the search strategy, and screening of abstracts was done to extract studies that met the inclusion criteria.

**Results:**

We retrieved 41 studies, of which 11 matched the inclusion criteria. Data were thematically arranged into studies measuring the validity and reliability of the CPAx tool, using the CPAx tool to measure outcomes in the ICU, the tool used at ICU and hospital discharge.

**Conclusion:**

The use of the CPAx tool has no impact on measuring the hospital length of stay or quality of life.

**Clinical implications:**

The tool is comprehensive and enhances the accuracy of patient assessment.

## Introduction

Patients admitted to intensive care units (ICUs) have severe or life-threatening injuries and illnesses (Vincent 2019). Patients in ICUs require constant care, close supervision by ICU clinicians, life support equipment and medication to restore bodily functions. These patients are usually immobile or sedated to prevent pain and anxiety (Griffiths & Hall 2010). Complications acquired in the ICU include intensive care unit-acquired weakness (ICU-AW), which may slow recovery and limit the patient from returning to their previous highest functional status (Griffiths & Hall 2010). Physiotherapists play a key role to prevent and manage complications acquired in the ICUs. Physiotherapy interventions include promoting lung function, early mobilisation and activity-focused rehabilitation (Holdar et al. 2019). Adequate tools are therefore needed to evaluate patients’ functional outcomes and the effect of the physiotherapy interventions.

In the ICU, various tools have been proposed to measure physical outcomes, and these data can then be used to assess and plan patient-specific rehabilitation programmes (Denehy et al. 2013). These include the Physical Function in Intensive Care Unit Test-scored (PFIT-s), the Perme Mobility Scale, the Surgical intensive care unit Optimal Mobilisation Score (SOMS), the ICU Mobility Scale (IMS), the Functional Status Score for the ICU (FSS ICU) and the Chelsea Critical Care Physical Assessment (CPAx) (Parry et al. 2015).

The CPAx tool was developed by Corner et al. in 2013. The CPAx tool is a non-invasive bedside measure used to holistically measure physical morbidity in the ICU (Corner et al. 2013). The CPAx tool assesses 10 items that include respiratory function, cough effect, movement in bed, supine to sitting on the edge of the bed, dynamic sitting balance, standing balance, sit to stand, transferring from the bed to the chair, stepping and hand grip strength. Each item is measured on a six‐point scale from level zero, representing total dependency, to level five, representing total independence. The CPAx tool can evaluate patients who are sedated, as well as those who are fully awake (Corner et al. 2013). In comparison with other tools used in the ICU, the CPAx tool can measure respiratory function in addition to strength and physical functions.

According to the evidence, the CPAx tool is more responsive in surgical patients. The objective of our study was to identify studies that used the CPAx tool to measure the physical function of adult patients nursed in an ICU. Our study also reviewed the validity and reliability of the CPAx tool.

## Method

An integrative review provided a summary of studies with various research designs to provide a comprehensive understanding of the use of the CPAx tool in an ICU. We followed Whittemore and Knafl’s (2005) framework for integrative reviews to conduct our review. To date, there are limited options available to holistically measure the physical function in ICUs in South Africa (Whelan, Van Aswegen & Corner 2018). The available tools generally assess only the patients’ function and muscle strength and do not track progression. The CPAx tool can monitor both the respiratory and physical functions as well as the progression thereof. This integrative review aims to identify studies that used the CPAx tool to measure the physical function of adult patients in an ICU.

### Literature search

We searched Google Scholar, Directory of Open Access Journals (DOAJ) and PubMed for articles. Keywords in the titles were used to identify articles. The search strategy included the use of Boolean operators such as ‘OR’ and ‘AND’ between keywords. Peer-reviewed articles included were published between 2013 and 2023. The CPAx tool was developed and first published in 2013. We included observational studies, quasi-experimental studies, an experimental study and clinometric studies. Keywords or search terms included the following: ‘Chelsea Critical Care Physical Assessment Tool’, ‘physiotherapy outcome measures in critically ill patients’ and ‘ICU outcome measures’. The first author screened the abstracts of the identified articles to identify studies that fit the inclusion criteria.

The inclusion criteria were as follows:

- Peer-reviewed articles published from 2013 to 2022.
- Evidence on functional outcomes measured in ICUs using the CPAx tool.

### Quality control

Data were collated using an integrative review approach to motivate the use of the CPAx tool to measure the physical functions in ICUs. The first author and a librarian searched for articles. We screened the titles and abstracts to identify articles that met the inclusion criteria. Included studies were appraised using the CASP Guidelines (Long, French & Brooks 2020). This tool has checklists designed for use with systemic reviews, randomised control trials, cohort studies, case-control studies, economic evaluations, diagnostic studies, qualitative studies and clinical predictions. Our review included observational studies as well as experimental studies as other studies did not fit the inclusion criteria.

### Data screening and extraction

From the included studies, the following data were extracted: the study title, the study method, the aim and setting of the study, the patient sample, the procedure and the results of the study. The information included in the review was analysed thematically.

## Findings

**FIGURE 1:**
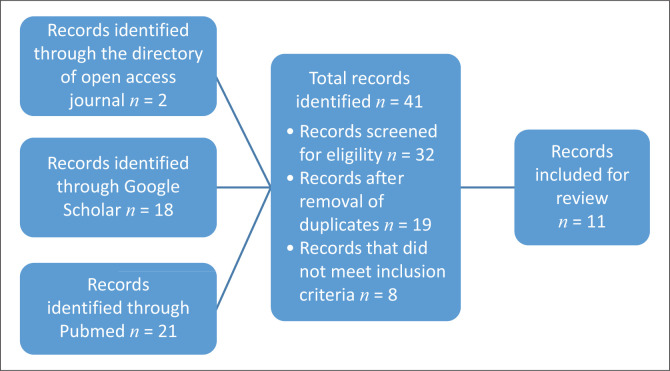
Flow diagram showing the selection of articles used in the integrative review Chelsea Critical Care Physical Assessment to measure the physical functions of patients in the intensive care unit.

Figure 1 illustrates the selection of studies. The search yielded 41 studies, 2 articles from the DOAJ, 18 from Google Scholar and 21 from PubMed. We screened 34 articles for eligibility. Nineteen studies remained after duplicates were removed. Eight of these studies did not fit the inclusion criteria. Eleven studies were included in the integrative review. The studies are summarised in Table 1.

**TABLE 1:** Data extracted from reviewed articles.

Authors (date)	Title	Study method	Aim, setting, sample	Methodology	Results
Astrup et al. 2018	Translation and cross-cultural adaptation of the Chelsea Critical Care Physical Assessment tool into Danish	Measurement property evaluation study	Aim: To translate the CPAx into Danish with cross-cultural validation of the translated version. Setting: A large interdisciplinary ICU of an academic hospital (Department of Intensive Care Medicine, Inselspital, Bern University Hospital, Switzerland) Sample: 30 patients in the ICU of the ages > 18 years	Step 1: CPAx translated to Danish. Step 2: Synthesis of results. Step 3: Back translation of the Danish CPAx to English and compared to the original version. Step 4: Three physiotherapists pre-tested the Danish CPAx on 30 ICU patients. Step 5: Focus group interview with three physiotherapists to evaluate cultural adaptation and applicability of the CPAx tool in clinical practice.	The CPAx is appropriate and applicable in clinical settings in Danish ICUs.
Corner et al. 2013	The Chelsea Critical Care Physical Assessment Tool (CPAx): validation of an innovative new tool to measure physical morbidity in the general adult critical care population; an observational proof-of-concept pilot study	Observational proof-of-concept pilot study	Aim: To develop a scoring system to measure physical morbidity in critical care – the Chelsea Critical Care Physical Assessment Tool Setting: Two London teaching hospitals Sample: 33 participants from trauma and general critical care unit	Focus group and observational study to test construct reliability against available measures such as the Glasgow Coma Scale Score, the Medical Research Council score to test muscle strength, peak cough flow, the Bloomsbury sedation score, the Australian Therapy Outcome Measures score, Sequential Organ Failure Assessment score, Short Form 36 (SF-36) score, days of mechanical ventilation and inter-rater reliability.	The CPAx demonstrated good content validity, correlated with available physical measures, and showed internal consistency and inter-rater reliability.
Corner et al. 2014	Construct validity of the Chelsea Critical Care Physical Assessment tool: an observational study of recovery from critical illness	Observational study	Aim: To evaluate the construct validity of the CPAx by analysing the association between CPAx scores as a measure of functional outcome and hospital-discharge location. Setting: An 11-bed ICU (mixed medical and surgical) in central London Sample: 499 patients of ages between 18 and 72 years admitted for 48 h to the ICU between 10 May 2010 and 13 November 2013	Patients were separated into seven categories at discharge. Descriptive statistics were used to assess the association between the ICU discharge CPAx tool score and hospital discharge location.	34.3% returned home with no ongoing rehabilitation or care input, four of these had CPAx = 50, 26.2% required community support, 5.6% went to inpatient rehabilitation for 6 weeks, 5.4% required nursing home level of care, 16.0% died in the ICU, 7.4% died in hospital. 3.2% had CPAx = 0, all of whom died within 24 h. A 0.8% ceiling effect and a 3.2% floor effect of the CPAx were found in the ICU. Compliance with completing the CPAx stabilised at 78% of all ICU admissions.
Corner et al. 2015	The responsiveness of the Chelsea Critical Care Physical Assessment tool in measuring functional recovery in the burns critical care population: an observational study	Observational study	Aim: To test the responsiveness of the CPAx tool in a Burns ICU. Setting: A two-bedded speciality Burns ICU in central London over a 31-month time period. Sample: 52 patients with a mean age of 47.1 years who were admitted for more than 48 h	All patients were assessed using the CPAx at pre-admission, ICU admission, ICU discharge, and hospital discharge. Analysis of variance, post-hoc between-group differences in median CPAx scores, and floor effect and ceiling effect for the four time points were completed. The minimal clinically important difference was estimated as half of the standard deviation of the CPAx score at ICU discharge.	*n* = 30 patients Mean age = 47.1 (s.d.: 21.2) 63.3% = men who sustained a median burn total body surface area (TBSA) of 30% (IQR: 11.3–48.8). Patients had significantly different CPAx scores at all four time points (*p* < 0.05). 86.7% of patients had full or zero CPAx pre-admission scores. For survivors, no patients scored full marks or zero on the CPAx at ICU discharge and at hospital discharge. On ICU admission, 66.7% scored zero on the CPAx, and no patients scored 50.
Eggmann et al. 2021	The German version of the Chelsea Critical Care Physical Assessment Tool (CPAx-GE): translation, cross-cultural adaptation, validity, and reliability	A prospective, single-centre, longitudinal, clinimetric study	Aim: To translate and cross-culturally adapt the CPAx to German (CPAX-GE) and to examine validity and reliability. Setting: The ICU of an academic hospital in Switzerland. Sample: 58 patients from an interdisciplinary ICU with more than 72 h of mechanical ventilation, age > 18 years, and sufficient language skills in oral and written German. The assessors were recently qualified and experienced physiotherapists. All physiotherapists were from Germany or Switzerland and spoke fluent German.	Explored the properties of the CPAx-GE in terms of construct, cross-sectional and cross-cultured validity. Relative reliability was analysed with intraclass correlation coefficients and absolute agreement was determined with the Bland-Altman plots. The CPAx was translated using a stepwise, forward-backward approach including cross-cultural adaptation with a multidisciplinary expert committee.	Validity was > 80% at baseline, in critical care, and at hospital discharge. The inter-rater reliability was high (ICC > 0.8) across all assessments. The limit of agreement ranged from −2 to 2 points. The error of measurement was small.
Eggmann et al. 2022	Predictive validity of the Chelsea Critical Care Physical Assessment tool (CPAx) in critically ill, mechanically ventilated adults: a prospective clinimetric study	Prospective clinimetric study	Aim: To investigate the predictive validity of the CPAx tool at ICU discharge in critically ill patients for their 90-day outcomes. Setting: A mixed ICU in an academic hospital in Switzerland. Sample: Critically ill adults aged >18 years who were mechanically ventilated for more than 72 h and in sufficient command of German; 60 patients were recruited of whom 50 had CPAx scores recorded at ICU discharge.	CPAx scores were recorded by certified physiotherapists at baseline between 72 and 144 h of ventilation, at ICU discharge, and hospital discharge. Therapists completed short, official online training. Demographic and hospital information was recorded. All participants were followed-up for 90 days after ICU discharge. A good outcome was defined as ‘residence at home 90 days after ICU discharge’. Non-survivors were identified from hospital databases. Survivors were contacted by phone and asked about their current residence, working status, and health-related quality of life.	CPAx at ICU discharge had good accuracy at predicting return to home within 90 days (AUC = 0.778). CPAx score significantly increased between discharge groups ‘undesirable’ vs. ‘rehabilitation’ vs. ‘home’ (*p* < 0.001). CPAx scores were not associated with 90-day health-related quality of life. Baseline CPAx scores correlated with length of ICU stay (*r* = −0.443).
Holdar et al. 2019	Cross-cultural adaptation and inter-rater reliability of the Swedish version of the Chelsea critical care assessment tool (CPAX-Swe) in critically ill patients	Observational study	Aim: To translate and culturally adapt the CPAx tool into Swedish. To test the inter-rater reliability of the CPAx-Swe in critically ill patients. Setting: ICUs and acute wards at the Karolinska University Hospital, Stockholm, Sweden Sample: 50 adult patients with a mean age of 56.8 years and a standard deviation of 18.9	The English CPAx was translated to the CPAx-Swe. A pilot test was done by two examiners who were not involved in the translation process to check if the CPAx-Swe was applicable to be used in acute Swedish healthcare settings. Assessments were done by 12 physiotherapists in pairs.	The CPAx-Swe was equivalent to the English CPAx. The pilot test showed the CPAx-Swe is applicable for clinical use. The CPAx-Swe had good reliability. Clinically, the CPAx-Swe proved to be a good assessment of function for patients in the ICU.
Whelan 2017	The use of the CPAx tool in a South African intensive care unit: clinical outcomes and physiotherapists’ perceptions	Part 1: A quasi-experimental design with a historically matched control group. Part 2: A survey-based design.	Aim: Does using the CPAx tool in the care of critically ill patients influence their clinical outcomes. To determine physiotherapists’ perceptions regarding the use of the CPAx tool in the care of critically ill patients. Setting: The medical, trauma and surgery ICUs at Chris Baragwanah Academic Hospital Sample: 26 participants with ages between 21 and 68 years; 14 (53.8%) underwent surgical procedures; 12 (46.2%) participants with traumatic orthopaedic injuries	Control group participants were matched with experimental group participants according to age, gender, diagnosis, acute physiology and chronic health evaluation (APACHE II) scores, CPAx scores, and sequential organ failure assessment (SOFA) scores were calculated for participants in the experimental group on alternate weekdays. ICU and hospital LOS were compared between the study participants, and a historical control group was done using the independent *t*-test. The relationship between CPAx, APACHE II, and SOFA scores was explored using Pearson’s correlation coefficients. Physiotherapists completed a questionnaire to determine their perceptions of the CPAx tool.	The CPAx tool proved to be more responsive in a surgical population than in a trauma population. Clinicians had positive perceptions of the CPAx tool for managing critically ill patients.
Whelan et al. 2018	Impact of the Chelsea Critical Care Physical Assessment (CPAx) tool on clinical outcomes of surgical and trauma patients in an intensive care unit: An experimental study.	Experimental study	Aim: To determine if the CPAx tool used as part of physiotherapy patient assessment, in two adult ICU settings where early patient mobilisation is part of standard physiotherapy practice, had an impact on ICU and hospital length of stay. Setting: Trauma and surgical ICU at the Chris Hani Baragwanath Academic Hospitalin South Africa Sample: 26 adult (ages between 27 and 44 years) participants admitted in the trauma and surgical ICU	Assessed the functional ability of participants every alternate day using the CPAx tool. The rehabilitation programmes were adjusted according to CPAx scores. The length of stay in ICUs and hospitals was noted and compared to the data of a matched historical control group.	Control group patients had significantly higher SOFA scores than patients in the intervention group (*p* = 0.005) (3.5 [IQR: 2–6.3] vs. 2 [IQR: 1.8–2.5]). Patients in the intervention group had median admission CPAx scores = 33.5 (IQR: 16.1–44) and median discharges = 38 (IQR: 28.5–43.8). ICU days and hospital length of stay were similar for both groups. Patients with lower CPAx scores at admission had longer hospital length of stay (*r* = −0.58, *p* = 0.00, *n* = 23). Patients with higher CPAx scores at discharge also have higher SOFA discharge scores (*r* = −0.58, *p* = 0.00, *n* = 23).
Wilson-Barry, Spencer & Haworth 2019	Feasibility for the use of the Chelsea Critical Care Physical Assessment tool in a complex neurorehabilitation unit	Longitudinal, non-experimental, correlational pilot study	Aim: To evaluate whether the CPAx tool is a sensitive and reliable measure of physical and respiratory function in neurorehabilitation inpatients. Sample: 29 adult patients admitted from the hospital for Level 1 rehabilitation following neurological injury in the United Kingdom.	CPAx scores were recorded by two physiotherapists on admission and at discharge. The UK version of the Functional Assessment Measure (UK FIM+FAM) is the principal outcome measure for specialist rehabilitation in patients with complex disabilities.	Inter-rater reliability was moderate to almost perfect for all items on CPAx. The strongest elements were lie-sit (ĸ = 0.960) and bed-chair (ĸ = 0.959); the weakest was cough (ĸ = 0.625). All linked dimensions of CPAx and the UK FIM+FAM were moderately correlated. High internal consistency between domains on CPAx and UK FIM+FAM (CPAx respiratory ɑ = 0.738, function ɑ = 0.935; UK FIM+FAM ɑ = 0.928). Floor effect was found for UK FIM+FAM for 68.75% of patients on admission and for 20.69% of patients on discharge. No floor or ceiling effect was seen on CPAx. Larger effect size on CPAx (*r* = 0.59) than UK FIM+FAM (*r* = 0.54).
Zhang et al. 2021	Chinesisation, adaptation and validation of the Chelsea Critical Care Physical Assessment Tool in critically ill patients: a cross-sectional observational study	Cross-sectional observational study	Aim: To translate and adapt the CPAx tool into Chinese (CPAx-Chi). To test the validity and reliability of the CPAx-Chi and to verify a cut-off point for diagnosing intensive care unit-acquired weakness (ICU-AW). Setting: General ICU of five third-grade class A hospitals in western China Sample: 200 critically ill adult patients (median age: 53 years; 64% men). Recruited participants were in the ICU setting for longer than 48 h and with a Glasgow Coma scale of more than 11.	Participants were assessed by two researchers individually and simultaneously using the Medical Research Council (MRC) Muscle Score and the CPAx-Chi.	CPAx items had a content validity index of 0.889. CPAx scale had a content validity index of 0.955. Compared to the MRC Score, the criterion validity of CPAx-Chi was *r* = 0.758 (*p* < 0.001) for researcher A, and *r* = 0.65 (*p* < 0.001) for researcher B. Inter-rater reliability was 0.902 (*p* < 0.001). The area under the receiver operating curve (AUC) of CPAx-Chi for diagnosing ICU-AW was 0.899 (95% CI: 0.862–1.025) and 0.874 (95% CI: 0.824 to 0.925) for researcher B.

CPAx, Chelsea Critical Care Physical Assessment; ICU, intensive care unit; IQR, interquartile range; s.d., standard deviation; CI, confidence interval; ICC, intraclass correlation coefficient; UK, United Kingdom.

### Themes

#### Theme 1: Validity

The validity of a scoring tool refers to the tool measuring what it is intended to measure. In the case of the CPAx, it was tested against different tools used and validated in ICU, to measure the functional outcomes (Corner et al. 2013). The CPAx assesses muscle strength, level of consciousness, cough effectiveness, and respiratory and physical functions (Corner et al. 2013). It was found to have a moderate to strong correlation with existing tools (Corner et al. 2013). The tool has been tested in critically ill patients who have sustained burns and traumatic injuries and in surgical populations showing improvements in physical function. The CPAx tool has also been translated into four other languages: Chinese, German, Swedish and Danish (Astrup et al. 2018; Eggmann et al. 2022; Holdar et al. 2019; Zhang et al. 2021). Construct and cross-sectional validity of the German version of the CPAx (CPAx-GE) are excellent with 86%, and the acceptance rate of the cross-cultural hypotheses based on the original CPAx is 83% (Griffiths & Hall 2010). Furthermore, the Chinese-translated CPAx also showed good content validity. The authors used nine ICU multidisciplinary experts with an expert authority coefficient that ranged between 0.75 and 0.95 (Zhang et al. 2021). Item-level index content validity (I-CVI) was from 0.889 to 1, and scale-level index content validity (S-CVI) was 0.955 (Zhang et al. 2021).

Construct validity shows the median and interquartile range of CPAx scores for patients when grouped by their discharge locations from the hospital (Corner et al. 2014). The analysis of variance shows statistically significant differences in the median CPAx scores among the seven discharge groups (H [2] = 311.4, *p* < 0.0001) (Whelan et al. 2018).

Predictive validity of the CPAx tool as an indicator of the functional prognosis of critically ill patients is found to be good (Corner et al. 2015). The criterion validity shows that the correlation coefficient between the MRC-Score and the CPAx-Chi ranges between 0.60 and 0.65 (Zhang et al. 2021) which is similar to what Corner et al. (2015) had found. The CPAx tool therefore has been shown to have good content validity.

#### Theme 2: Reliability

The next theme reviewed was the ability of the CPAx tool to produce consistent results, that is, reliability. The CPAx is found to be reliable by the original authors as well as in the translated versions. The tool proves to have internal consistency and inter-rater reliability with a κ = 0.988 and α = 0.798 (Corner et al. 2013). Cronbach’s α for CPAx-Chi is 0.939, and the inter-rater reliability is 0.902 when the original CPAx is translated (Zhang et al. 2021). The inter-rater correlation coefficient is > 0.8 for the items of respiratory function, transfer from bed to chair and grip strength. The inter-rater correlation coefficients of other items of CPAx-Chi are all > 0.7 (Zhang et al. 2021). The inter-rater reliability of the CPAx tool is moderate to almost perfect for most of the components in relation to other outcome measures with the strongest elements being lying to sitting and mobility, from the bed to the chair, with cough having the weakest inter-rater reliability (Wilson-Barry et al. 2018).

Inter-rater reliability by observation is excellent with intraclass correlation coefficient (ICC) > 0.8 including 95% confidence interval (CI) on patient assessment with the translation of the German CPAx (Eggmann et al. 2021). The constructed Bland–Altman’s plots confirm the high agreement of the CPAx-GE with a mean difference of 0.13 ± 0.15 (95% limit of agreement: −2.04 to 1.79) (Eggmann et al. 2021). The inter-rater reliability of the Swedish CPAx tool (CPAx-Swe) is found to be satisfactory and applicable for use within the clinical setting (Holdar et al. 2019). Reliability of the aggregated scores and the individual items is found to be good (Holdar et al. 2019). The ICC of 0.97 and the quadratic weighted kappa values range from 0.88 to 0.98 (Holdar et al. 2019).

#### Theme 3: An outcome measure in the intensive care unit

Various studies reported that the CPAx tool is useful for assessing the physical function of critically ill patients in an ICU (Astrup et al. 2018; Holdar et al. 2019; Whelan 2017). The tool shows a high consistency with measuring respiratory and functional outcomes in different ICUs. The CPAx is shown to be a good measure of clinical progress in the patient’s functional status after they sustained burn injuries (Corner et al. 2015). Corner et al. (2015) reported that a change in CPAx score of six or more can be considered a clinically meaningful change in physical function.

The length of stay (LOS) in ICU and in hospital is not significantly influenced by the addition of the CPAx tool to standard physiotherapy patient management although physical function improves (Whelan et al. 2018). Whelan and colleagues noted that therapists find that the CPAx tool enhances their accuracy of patient assessment in the ICU setting. They reported that the CPAx assists with patient care and planning, assists with the evaluation of patient progression, serves as motivation for patients to participate in treatment, enhances communication with patients and motivates them regarding patient response to treatment (Whelan et al. 2018).

#### Theme 4: Measurement at discharge from the intensive care unit and hospital

The CPAx scores correspond well with the discharge destination of patients. Patients with higher CPAx scores can go home post-discharge (Corner et al. 2014). Importantly, CPAx scores measured at baseline are found to correlate with the LOS in critical care units (Eggmann et al. 2022).

As far as changes in the score during ICU stay, the difference in median CPAx scores between ICU admission and discharge is 4.5 (Whelan et al. 2018). From ICU admission to discharge, the median CPAx scores changed by 3.2 points for the surgical group and by 7.5 points for the trauma group in terms of functionality.

In terms of correlation of the CPAx scores on ICU admission to hospital LOS, a moderate negative correlation is shown (*r* = −0.58, *p* = 0.001, *n* = 23). There is, however, no correlation between CPAx at admission and ICU LOS (*r* = −0.19, *p* = 0.38, *n* = 23) or between CPAx at ICU discharge and ICU LOS (*r* = −0.58, *p* = 0.13, *n* = 8) or hospital LOS (*r* = −0.11, *p* = 0.78, *n* = 8) (Whelan et al. 2018).

## Discussion

The CPAx tool is shown to be a good measure of physical function for patients admitted in the ICU presenting with different conditions. The tool can assess physical limitations upon discharge from the ICUs to discharge locations. The tool also assists in planning patient-specific rehabilitation (Whelan et al. 2018). The CPAx tool, however, does not predict the LOS of a patient in the hospital. The CPAx tool is shown to have good internal consistency and inter-rater reliability. The tool has high consistency with assessing functional outcomes as well as respiratory functions in patients nursed in the critical care setting.

Clinicians agree that the tool assists with assessing patients in an ICU and that the CPAx tool could be used to plan patient-specific rehabilitation goals. In the identified studies, the physical function of patients improves between admission and discharge from the ICU. The CPAx tool identifies improved functional outcomes in both surgical and trauma patients. The tool assists with developing patient-specific treatment plans that indeed improve physical functions (Whelan et al. 2018). Participants in the surgical group have significantly better physical function at ICU discharge in comparison to trauma patients. Even though physical function improves, there is no impact on the LOS in both ICUs and hospitals (Whelan et al. 2018).

Schaller et al. (2016) noted that improvement in functional outcome may be quicker for patients who are more awake and alert and respond favourably to medical care and rehabilitation care provided to them while in the ICU and hospital. As their cooperation improves and their condition stabilises, patients are more likely to participate in functional activities (Whelan et al. 2018). Indeed, the German CPAx (CPAx-GE) at ICU baseline is mainly determined by respiratory function and movement, while at ICU discharge, basic activities start to emerge, and at hospital discharge, standing, transferring and stepping became more practised for patients (Eggmann et al. 2021).

Assessments done using the tool assist in identifying the impairments and patient activity limitations. The CPAx tool as an outcome measure assists physiotherapists in planning individual patient-specific rehabilitation programmes to better serve patients. The supporting studies were limited to trauma and surgical ICUs. The tool is a supported measure of physical morbidity in patients admitted to an ICU setting with further research recommended in different ICUs and with a bigger study population.

## Conclusion

The CPAx tool is a good measure of physical function and respiratory function for patients admitted to an ICU. The CPAx tool, however, does not have an impact on the LOS within the ICU and the hospital, nor does it predict health-related quality of life. Studies have only assessed patients in ICUs and not in a high-care setting that forms part of the critical care setting. The CPAx tool is comprehensive, enhances the accuracy of patient assessment and assists physiotherapists to draw up patient-specific treatment plans to address the identified impairments.
